# Impact of Extending Hard-Cheese Ripening: A Multiparameter Characterization of Parmigiano Reggiano Cheese Ripened up to 50 Months

**DOI:** 10.3390/foods9030268

**Published:** 2020-03-02

**Authors:** Paolo D’Incecco, Sara Limbo, John Hogenboom, Veronica Rosi, Serena Gobbi, Luisa Pellegrino

**Affiliations:** Department of Food, Environmental and Nutritional Sciences (DeFENS), Università degli Studi di Milano, 20133 Milan, Italy; sara.limbo@unimi.it (S.L.); john.hogenboom@unimi.it (J.H.); veronica.rosi@unimi.it (V.R.); serena.gobbi@unimi.it (S.G.); luisa.pellegrino@unimi.it (L.P.)

**Keywords:** cheese ripening, ripening extension, cheese microstructure, free amino acids, capillary electrophoresis, proteolysis, volatile compounds, confocal laser scanning microscopy

## Abstract

Extending ripening of hard cheeses well beyond the traditional ripening period is becoming increasingly popular, although little is known about the actual evolution of their characteristics. The present work aimed at investigating selected traits of Parmigiano Reggiano cheese ripened for 12, 18, 24, 30, 40 and 50 months. Two cheeses per each ripening period were sampled. Although moisture constantly decreased and was close to 25% in 50-month cheeses, with a parallel increase in cheese hardness, several biochemical changes occurred involving the activity of both native and microbial enzymes. Capillary electrophoresis demonstrated degradation of α_s1_- and β-casein, indicating residual activity of both chymosin and plasmin. Similarly, continuous release of free amino acids supported the activity of peptidases deriving from lysed bacterial cells. Volatile flavor compounds, such as short-chain fatty acids and some derived ketones, alcohols and esters, evaluated by gas chromatography with solid-phase micro-extraction, accumulated as well. Cheese microstructure was characterized by free fat trapped in irregularly shaped areas within a protein network, with native fat globules being no longer visible. This study showed for the first time that numerous biochemical and structural variations still occur in a hard cheese at up to 50 months of aging, proving that the ripening extension deserves to be highlighted to the consumer and may justify a premium price.

## 1. Introduction

Parmigiano Reggiano is an Italian extra-hard cheese made from raw milk. Being a protected designation of origin (PDO) product, it is produced in a restricted geographical area using the traditional cheesemaking described in the product specification [1]. Milk is partly skimmed by natural creaming, poured into the traditional open copper vat with natural whey starter and calf rennet in order to achieve coagulation at 33–34 °C within 8–10 min. The curd is cut into rice-grain-sized granules while the temperature is progressively raised up to 53–54 °C in 10–12 min under gentle stirring. When stirring is stopped, the curd granules sediment and aggregate at the bottom of the vat due to the compression that hot whey exerts for about an hour. The curd is then extracted from the vat, cut into two portions that are set into circular molds and kept there to slowly cool down, acidify and lose more whey for about two days. Afterward, cheese wheels (~35 kg each) are salted in brine for 20–22 days and then ripened in a ripening room at about 18 °C and 80% relative humidity. The minimum ripening period for PDO Parmigiano Reggiano is 12 months.

It is commonly recognized that quality of artisanal cheeses mostly relies on the skill of the cheesemaker to master the vat process by day-to-day adapting the extent and timing of the different actions to the characteristics of the raw materials, namely raw milk and the natural starter culture. Nevertheless, the typical cheese characteristics develop during the subsequent ripening period, when a variety of profound and complex changes take place. These changes imply phenomena that are particularly interesting in long-ripened cheeses made from raw milk, such as Parmigiano Reggiano and Grana Padano, and that have been addressed in many research studies and review articles [2,3].

Enzymes such as the native plasmin and the rennet chymosin, both largely retained in the curd, soon attack casein fractions, initiating the proteolytic maturation of cheese. Further degradation of casein into peptides [4] and free amino acids [5] takes longer and needs the contribution of microbial peptidases. Microbial lipases are responsible for the release of free fatty acids that contribute to cheese flavor development [6]. Both proteolysis and lipolysis products proved to reach characteristic profiles in raw milk PDO cheeses at different ages [6,7], confirming that the microbial populations are rather homogeneous within the respective production areas and act in a repeatable way because the selective action of both the cheesemaking and ripening conditions are markedly consistent among cheese factories.

A variety of microbial populations are natural contaminants of raw milk, including the so-called nonstarter lactic acid bacteria (NSLAB), whereas others are added with the natural starter, predominated by lactic acid bacteria. Different populations are active or inactive throughout ripening, depending on the selectivity of environmental conditions that are progressively changing within the cheese. When starter lactic acid bacteria (SLAB) decline, NSLAB rise and largely contribute to cheese flavor development, as mesophilic facultatively heterofermentative species dominate in this population [2,8].

During the recent years, increasing amounts of hard cheeses such as Parmigiano Reggiano, Grana Padano, Comté, Cheddar or Gouda, are kept ripening for longer periods than usual in the past. Considering that consumption rates are globally increasing for those cheeses and new market areas are presently accessible, it is of interest to the manufacturers to provide the consumers with a wider array of products, including cheeses having more pronounced taste and brittle texture. To date, there has been little research focus on cheese ripening periods over 24 months [5,9].

In this study, we tracked various composition, structure and appearance characteristics of Parmigiano Reggiano cheeses during a ripening period up to 50 months, in order to elucidate whether some of these are still evolving over such a long period and might be useful in characterizing the longer-aged cheeses. Regardless of the distinctive sensory properties that are well known and appreciated by the consumers worldwide [10], we were interested in estimating the significance of extending the ripening of Parmigiano Reggiano cheese far more than the usual 12–18 months. Therefore, different aspects were addressed and a wide array of analytical parameters were tested on cheeses ripened from 12 to 50 months.

## 2. Materials and Methods

### 2.1. Cheese Samples

**Figure 1:**
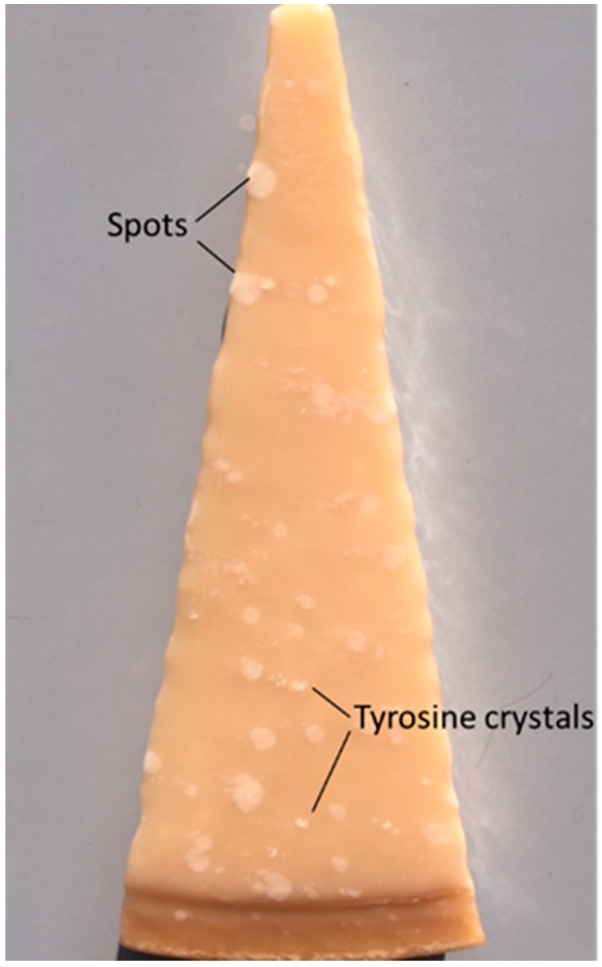
Slice of 40-month ripened Parmigiano Reggiano cheese.

A total of 12 Parmigiano Reggiano (PR) cheeses were obtained from six factories located in the production area of the PDO Parmigiano Reggiano cheese. Two cheeses of a specific ripening period were obtained per factory. The manufacture and ripening conditions provided by PDO Parmigiano Reggiano specification and described in Figure S1 were followed. Cheese ages were 12 (12mo), 18 (18mo), 24 (24mo), 30 (30mo), 40 (40mo) and 50 months (50mo) with ±1 month of incertitude. From each cheese wheel, two vertical slices (around 1 kg each) were taken. One slice was used to count white spots and tyrosine crystals visible on the surface (100 cm^2^) as described by D’Incecco et al. [11] (Figure 1) and then used for color, rheology and microscopy analyses that were immediately conducted in sequence in order to avoid cheese drying. The other slice was finely grated using a domestic grinder, after removal of 0.5-cm rind layer, and submitted to chemical analysis within the next day.

### 2.2. Cheese Composition Analysis

Cheese samples were analyzed for moisture, fat and protein content using the International Standard methods of the International Dairy Federation, as described previously [7]. Protein content was calculated using 6.38 as conversion factor. The moisture in the nonfat substance (MNFS) was calculated as 100 × moisture content/(100−fat content). Analyses were carried out in duplicate.

### 2.3. Proteolysis Extent

#### 2.3.1. Casein and Peptides

**Figure 2:**
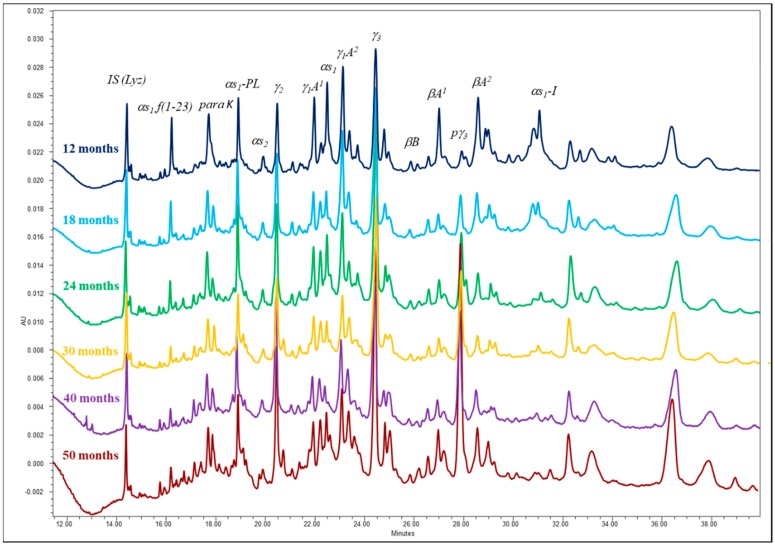
Capillary electrophoresis patterns of Parmigiano Reggiano cheeses ripened up to 50 months.

Intact casein fractions and major peptides were analyzed by capillary zone electrophoresis (CZE), adopting the conditions described by D’Incecco et al. [12]. Grated cheese (1 g) was dispersed in a sample buffer (10 mL) and kept at room temperature for at least 4 h. Sample buffer was prepared by adding 200 mL of 60% (*w/v*) urea solution in Millipore MilliQ purified water and 300 mL of urea 60% (*w/v*)/methylhydroxyethylcellulose (MECH) 0.15% (*w/v*) in MilliQ water with 7.44 g of ethylenediaminetetraacetic acid disodium salt dihydrate, 6.06 g of tris(hydroxymethyl)aminomethane, 2.64 g of 3-(N-morpholino)propanesulfonic acid and 0.77 g of dithiothreitol. The sample was further diluted 1:5 with the same buffer, filtered with 0.22 µm polyvinylidene fluoride membrane filter (Millipore) and separated using a hydrophilically coated capillary column (50 µm i.d., 0.05 µm coating, 500 mm effective length, 100 × 800 µm slit opening, DB-WAX 126-7012, J&W Agilent Technologies, Milan, Italy). Separation was carried out at 45 °C with linear gradient from 0 to 30 kV in 4 min, followed by constant voltage at 30 kV for 36 min, using P/ACE^TM^ MDQplus capillary electrophoresis equipment (AB Sciex, Milan, Italy) including a UV detector set at 214 nm. Separation buffer was prepared as follows: 60 mL of urea 60% (*w/v*)/MECH 0.15% (*w/v*) in MilliQ water solution were added with 4.38 g of citric acid monohydrate, 0.59 g of trisodium citrate dehydrate and 40 mL of MilliQ water. Separation buffer was filtered with 0.45 µm regenerated cellulose membrane filter (Agilent Technologies). Peak identification in the obtained electropherograms is shown in Figure 2. The peak area ratios between selected casein or peptide fractions were calculated, considering normalized peak area (peak area counts/migration time), as follows:

(1) $$\frac{\alpha_{s1}-I}{\alpha_{s1}}=\frac{\alpha_{s1}-\mathrm{CN}-I\;8P}{\alpha_{s1}-\mathrm{CN}\;8P}$$

(2) $$\frac{\alpha_{s1}f\left(1-23\right)}{\alpha_{s1}}=\frac{\alpha_{s1}-\mathrm{CN}\;f\left(1-23\right)}{\alpha_{s1}-\mathrm{CN}\;8P}$$

(3) $$\frac{\Sigma \gamma }{\Sigma \beta }=\frac{\gamma 1-\mathrm{CN}\;A1+\gamma 1-\mathrm{CN}\;A2+\gamma 2-\mathrm{CN}\;A+\gamma 3-\mathrm{CN}\;A+p\gamma 3-\mathrm{CN}\;A}{\beta -\mathrm{CN}\;B+\beta -\mathrm{CN}\;A1+\beta -\mathrm{CN}\;A2}$$

where α_s1_-CN 8P is α_s1_-CN with eight phosphorylated serine residues.

Cheese age, expressed in months, was calculated according to Masotti et al. [5] as follows:

(4) $$\mathrm{Cheese}\;\mathrm{age}=0.91\left(100\frac{p\gamma_{3}-\mathrm{CN}\;A}{p\gamma_{3}-\mathrm{CN}\;A+\gamma_{3}-\mathrm{CN}\;A}\right)+4.33$$

where pγ_3_-CN is the pyroglutamyl-γ3-casein.

Analyses were carried out in duplicate.

#### 2.3.2. Free Amino Acids

Ion-exchange chromatography was used for free amino acid (FAA) analysis following the conditions described by Hogenboom et al. [13]. The equipment was an amino acid analyzer Biochrom 30plus (Biochrom Ltd., Cambridge, UK). Free amino acids were extracted from grated cheese (1.5 g) previously dissolved in 0.2 N sodium citrate buffer, homogenized then deproteinated with 7.5% 5-sulfosalicylic acid. The obtained solution (10 mL) was added with 2 mL of a 600 mg/L solution of Norleucine as an internal standard and diluted in 0.2 N lithium citrate. Extracts were filtered on 0.2 µm cellulose acetate filter (Millipore) prior to injection. The chromatographic conditions were those recommended by the manufacturer. A multipoint calibration was used for quantitation of 21 amino acids. Analyses were carried out in duplicate.

### 2.4. Volatile Compounds

The determination of volatiles was carried out on the samples with different ages. Five grams of grated cheese was weighted in a 20 mL glass vial sealed with an aluminum cap provided with a silicon septum (HTA, Brescia, Italy). A carboxen-polydimethylsiloxane-divinylbenzene (CAR-PDMS-DVB; 50/30 µm × 1 cm) (Supelco, Bellefonte, PA, USA) was used to collect volatiles from the samples using an automatic SPME autosampler (HTA, Brescia, Italy) set at the following conditions: incubation for 10 min at 40 °C; agitation for 5 min; extraction for 45 min; desorption for 20 min. After the extraction step, the volatiles were release in the injector of a gas chromatograph (Perkin Elmer Autosystem XL Gas Chromatograph) coupled with a mass spectrometer (Turbomass, Perkin Elmer, Italy). The injector was set at 250 °C and the injection mode was splitless for 0.50 min. The gas-chromatographic separation was carried out with a Stabilwax-MS column (30 m × 0.250 mm × 0.25 µm; Restek, Bellefonte, PA, USA) using helium as carrier at flow rate of 1.2 mL/min. The oven temperature was initially set at 40 °C and held for 8 min, ramped at 4 °C/min up to 220 °C and held for 15 min. The transfer line temperature was set at 200 °C and the source temperature at 250 °C. The mass spectrometer operated in electron ionization mode at 70 eV using the full scan mode. The MS detector registered the *m*/*z* in the range from 35 up to 350 Da. The ions used for identification were chosen according to the National Institute of Standards and Technology (NIST) MS Search 2.0 library and validated by external standard comparisons of ion fragmentation patterns and by calculating the linear retention index (LRI) running an alkane standard solution (C8–C20, Merck, Italy). Values were expressed as area units/10,000. Triplicate injections were carried out for each cheese sample.

### 2.5. Cheese Color

Color analysis was carried out on cheese slice surface using a portable tristimulus colorimeter (Minolta Chroma Meter CR 300—Minolta, Osaka, Japan), equipped with an 8 mm viewing port, illuminant C source and standard observer. Color coordinates (L*, a*, b*) were measured in triplicate on each cheese slice and different color indexes were calculated [14]. Yellowness index (YI) [15] was calculated as:

(5) $$\mathrm{YI}=142.86\frac{b^{*}}{L^{*}}$$

Total color difference (ΔE*) between the color index recorded at each ripening time and that recorded at 12 months was obtained from the equation:

ΔE* = (Δa*^2^+Δb*^2^+ΔL*^2^)^1/2^ (6)

Hue angle (h*) was calculated as:

(7) $$h^{*}=\tan^{-1}\;\left(\frac{b^{*}}{a^{*}}\right)$$

### 2.6. Cheese Texture Analysis

Texture was analyzed using a TA-XT Plus (Stable Micro System, Surrey, England) texture analyzer equipped with the fracture wedge set comprising upper and lower wedges with cutting angle of 30° and 30 mm width. The upper wedge was connected directly to the load cell. The wedge fracture test was carried out on three cheese portions (2 cm × 2 cm × 2 cm) that were cut from each cheese slice at 5-cm depth below the rind of the round side and half-height of the cheese and conditioned at room temperature before analysis. Penetration was performed at a constant crosshead speed of 1 mm/s until either 70% of height or the fracture of the cheese cube was reached. The force/time curves were used to calculate cheese hardness (N) and the fracture deformation or brittleness (mm) according to manufacturer guidelines.

### 2.7. Cheese Microstructure

Microstructure of cheese samples was analyzed by Confocal Laser Scanning Microscopy (CLSM) as described previously [16]. Three portions of cheese (around 2 mm × 2 mm ×1 mm) were taken from each cheese slice at 5-cm depth using a razor blade. Samples were stained with Nile Red (Sigma Aldrich, St Louis, MO, USA) and Fast Green FCF (Sigma Aldrich) to visualize fat and protein matrix, respectively. The staining was carried out as follows: the stock solutions of Nile Red (1 mg/mL in dimethyl sulfoxide) and Fast Green (1 mg/mL in Millipore MilliQ purified water) were diluted tenfold just prior to 5-min staining. Samples were analyzed by using an inverted confocal laser scanning microscope A1+ (Nikon, Minato, Japan). The excitation/emission wavelengths were set at 488 nm/520–590 nm for Nile Red and at 638 nm/660–740 nm for Fast Green FCF [17]. Images are presented as maximum projection of 23 optical sections stacked together with separation between layers set at 0.30 μm. Image analysis was performed using Vision4D software (Arivis, AG, Germany) on maximum projection of CLSM z-stack images. Porosity was calculated as the ratio between the nonfluorescent volume (µm^3^) and the total fluorescent volume (µm^3^).

### 2.8. Statistical Analysis

The data of composition, FAA, structure, rheology and color were assessed by one-way analysis of variance (ANOVA) and significant differences were considered at *p* < 0.05 level as evaluated by Tukey’s test using SPSS Win 12.0 program Version 22 (SPSS Inc. IBM Corp., Chicago, IL). Principal component analysis (PCA) was carried out on selected variables showing a specific trend with respect to ripening time. Before PCA, data were preprocessed using the auto-scale mode and transformed using the normalized method. The Unscrambler v.9.7 software (Camo Software AS, 2007, Oslo, Norway) was used. Differences at *p* < 0.05 (*); *p* < 0.01 (**) and *p* < 0.001 (***) were considered significant.

## 3. Results and Discussion

### 3.1. Composition Analysis

The gross composition of the PR cheese samples of different ages is shown in Table 1. As expected, moisture was the most intensively changing among cheese components. Our data showed a progressive decrease during the considered period, although the variability of mean values was quite high in some cases. Values as low as 25 g/100 g cheese were reached in the 50mo ripened cheeses, which were markedly lower than the moisture content of 27 g/100 g measured by Malacarne et al. [18] in a 54-month old PR cheese. Fox et al. [19] attributed the maintenance of Parmesan cheese quality to its low levels of both moisture content (29.2%) and water activity (0.917). The protein content of the cheeses roughly varied from 31.2 and 33.8 g/100 g, with no characteristic trend over the ripening time. Concomitantly, the fat content was unusually variable (29.2–35.7 g/100 g) and the range of values on dry matter basis was wide as well (42.1–47.9 g/100 g). A high fat content makes cheese structure softer, especially when the moisture content is low [20,21]. Likely, when making cheeses destined to a prolonged ripening, the cheesemakers intentionally remove less fat from raw milk by shortening the creaming time in order to achieve this effect. To exclude the variability of fat content on cheese moisture, MNFS can be considered. Indeed, this parameter showed that the proportion of moisture decreased throughout the whole ripening period. Cheeses with MNFS < 51% are classified as extra-hard by Codex Alimentarius [22].

### 3.2. Proteolysis

The extent of primary proteolysis was evaluated by CZE as the extent of casein fraction degradation (Figure 2). The CZE patterns evidenced very extensive changes taking place in PR cheese during the whole prolonged ripening period considered in this study and, nevertheless, many peaks corresponding to both intact casein and large fragments were still present in the most aged samples. Besides the specific cleavage of k-CN, rennet chymosin typically cleaves α_s1_-CN at Phe_23_-Phe_24_, splitting the protein chain into two fragments, i.e., α_s1_-CN f(1-23) and f(24-199), also called α_s1_-I-CN. A progressive decrease of both α_s1_-CN and the main derived fragments occurred throughout the whole ripening period (Figure 2). The peak-area ratio between these two fragments and the intact α_s1_-CN slightly decreased over time, indicating that the degradation of the former proceeded further (Table 2). To our knowledge, no direct evidence is available in the literature of persistence of chymosin activity in long-ripened cheeses. It has been shown that, in high-temperature cooked cheeses, chymosin is inactivated [23,24] and the main α_s1_-CN-derived fragments accumulate during the first 4-6 months of ripening. In contrast to this current view, Hynes et al. [25] demonstrated that in laboratory-manufactured Reggianito Argentino cheese cooked at 52 °C, not at 60 °C, chymosin partly reactivated during ripening, and this recovered activity contributed to α_s1_-CN degradation. The authors, however, prolonged their observation over 3 months only. Considering that conditions other than cooking temperature [23,24] also concur in chymosin retention in the curd and inactivation, it can be reasonably assumed that a low quantity of residual chymosin could proceed to slowly degrade casein, likely in a localized manner.

Plasmin is reported to be a major proteolytic enzyme in cooked cheeses [24,26], with β-CN being its primary substrate. This last is progressively degraded into the ɣ-CNs which, on the contrary, are rather stable and accumulate [4,23]. The ratio between ɣ-CNs and β-CNs (Σγ/Σβ) progressively increased by a factor of 4.5 in the ripening time from 12 to 50 months (Table 2), suggesting that plasmin activity proceeded. Consistent with these findings, Mayer et al. [26] showed that β-CN was no longer detectable by gel electrophoresis in a 48-month aged PR, while it was in less aged cheeses. In a previous study, we proposed the pyroglutamyl-ɣ_3_-CN (p-ɣ_3_-CN) as an indicator of cheese age, which proved to be accurate for both Grana Padano and PR cheeses [5]. This peptide originates from the cyclization of the N-terminal residue of glutamic acid of ɣ_3_-CN and proved to be highly stable to further proteolysis during cheese ripening. In the present study, we have evaluated the actual cheese age of the PR samples using this approach and found values pretty close to those declared by manufacturers (Table 2). This finding strongly supports the persistent plasmin activity, since ɣ_3_-CN increased progressively and therefore the precursor of p-ɣ_3_-CN was freely available.

Secondary proteolysis, typically operated by bacterial enzymes, is well described by free amino acids (FAA) since they are the final products of enzymatic splitting of peptones and peptides [23]. Surprisingly, the content of FAA still significantly increased in PR cheeses from 12 to 50 months, when utilization by LAB is expected to be over, suggesting that proteases released by lysed cells can still have a role (Table 2). However, the limited number and variable origin of the samples considered in this study did not allow to reveal differences in FAA content in cheeses with similar age. Nevertheless, our data indicated that, in such long-ripened cheeses, approximately one-third of the protein is present in a directly utilizable form for humans. To the knowledge of the authors, no cheese types other than PR and Grana Padano display such high FAA content.

As previously observed in other cheese types [7,19,20], glutamic acid (Glu) was by far the most abundant among FAA (Table S1). Besides being split from proteins and peptides, Glu may also derive from glutamine (Gln) deamidation by glutaminase and, in turn, can be decarboxylated to y-aminobutiric acid (GABA) by glutamate decarboxylase. Both Gln deamidation and Glu decarboxylation are involved in acid-resistance mechanisms identified in selected LAB [27,28,29]. The involved enzymes are more abundant in the cytoplasm and therefore can be active after cell lysis [23]. Indeed, almost no Gln was detected in PR cheeses ripened for 30 months or longer. The same pattern was found in long-ripened Grana Padano cheese [5]. Accumulation of aspartic acid (Asp) was slower, compared to that of Glu, and a smaller concentration of Asp was found in the 50mo PR cheeses. Although scarcely documented, Asn deamidation activity to form Asp was observed in selected LAB starters, especially facultatively heterofermentative species [30].

### 3.3. Volatile Compounds

The composition of the volatile compounds (VOCs) of PR cheeses ripened from 12 up to 50 months and detected by SPME-GC-MS is compiled in Table 3, where the identified substances are listed. Short-chain odd-numbered free fatty acids (FFA), esters and ketones were the most abundant compounds and accounted for 86%, 7% and 6% of the total area, respectively.

The total volatile fraction roughly tripled across the considered ripening period, particularly because of the increased amount of short-chain FFA, as similarly observed by Malacarne et al. [6] in PR cheese during 24-month ripening. To the best of our knowledge, no studies investigated the relationship between the autolysis of mesophilic NSLAB and the levels of volatile compounds in cheese at such long ripening times. However, the presence of free fat in large areas embedded in the protein network, as was observed by CLSM in our cheese samples (see Section 3.4), is a condition that may largely favor enzyme activity towards triglycerides.

In fact, in extra-hard cheeses, an extensive lipolysis of triglycerides can occur, supported by both microbial and native milk enzymes like lipases and esterases, and the FFA produced can directly or indirectly contribute to cheese aroma development. Esterases responsible for hydrolyzing short acyl ester chains (C2–C8) are intracellular enzymes, and those from mesophilic nonstarter lactobacilli (NSLAB) are reported to be the main contributors to short-chain FFA accumulation during ripening of PR cheese [2]. Lipases hydrolyze longer acyl ester chains that are characterized by more than 10 carbons [31].

As shown in Table 3, butanoic, hexanoic and octanoic short-chain fatty acids represented the main volatile acids, and their contribution increased during ripening. Butanoic acid is responsible for buttery–cheesy flavor, hexanoic acid for sweaty and sometimes pungent flavor and octanoic acid for goat-like flavor [32]. Qian and Burbank [33] indicated odor activity values (OAVs, i.e., the ratio between the concentration and the flavor threshold) equal to 0.5, 28 and 320 for octanoic, hexanoic and butanoic acid, respectively, and together the three FFA mainly contributed to the typical aroma of PR cheese. Few branched FFA were identified, especially at the beginning of the ripening (18–24 months): 2-methyl butanoic acid and, to a greater extent, 3-methyl butanoic acid. These branched FFA have similar aroma characteristics as the corresponding linear FFA, and they are characterized by a lower threshold because of their higher vapor pressure [33]; therefore, they can subtly contribute to the overall flavor of PR cheese.

Short- and medium-chain FFA themselves contribute to the aroma while other FFA are precursors for the formation of other flavor compounds, such as methyl ketones, lactones, esters, alkanes and secondary alcohols [23]. The presence of these secondary volatile compounds plays an important role in enhancing cheese flavor complexity [34]. In PR cheese, ethyl butanoate, ethyl hexanoate and ethyl octanoate were detected, and their presence tended to increase during ripening. These esters have strong fruity and floral notes [35] and are primarily responsible for the fruity aroma in PR cheese due to their very low sensory threshold (on the order of 0.04–0.6 mg/kg fat) and high OAV [33]. Most of these compounds were already recognized in hard raw-milk cheeses. A predominance of both FFA and ketones among VOCs was also found by Abbatangelo et al. [36] in PR cheese, by Lazzi et al. [37] in 13-month Grana Padano cheese and by Ceruti et al. [38] in Reggianito cheese.

Among ketones, 2-heptanone was the most abundant ketone identified in our samples at all ripening times, with even higher amounts in longer ripened ones. In particular, 2-heptanone, together with other compounds, was identified among the most important compounds responsible for the characteristic aroma of PR with its fruity and moldy flavor contribution [33]. Ketones can be further transformed in secondary alcohols, and we identified 2-pentanol and 2-heptanol at all ripening times. Even if secondary alcohols are thought to poorly contribute to cheese aroma, 2-heptanol was identified as a key odorant in Grana Padano cheese [39]. Overall, the prolongation of the ripening up to 50 months favored the enrichment of volatile compounds that give the typical aroma to PR cheese, without inducing changes that could generate off-flavors.

### 3.4. Cheese Microstructure, Appearance and Texture

**Figure 3:**
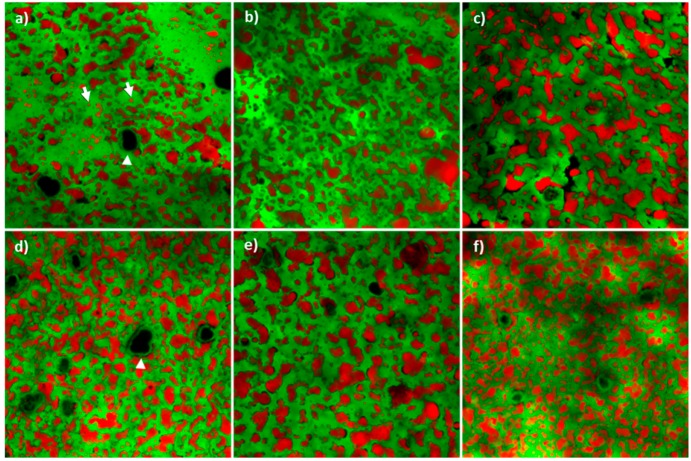
Confocal microscopy images of 12 months (**a**), 18 months (**b**), 24 months (**c**), 30 months (**d**), 40 months (**e**) and 50 months (**f**) ripened Parmigiano Reggiano cheeses. Intact fat globules (white arrows, panel a) were mostly observed in 12-month ripened Parmigiano Reggiano cheese. Calcium phosphate crystals (white arrowheads, panels **a** and **d**) were present at all ripening times.

Regardless the cheese ripening stage, fat was confined in irregular globular-shaped areas trapped within the protein network, suggesting these to originate from coalescence of fat globules of different size (Figure 3). Coalescence evolved during ripening, as confirmed by the lower number of fat globules in most aged cheeses (Table 4), and some small intact globules were detected in the 12mo cheeses only (Figure 3a, arrows). Other parameters, such as sphericity and fat volume, were not significantly affected by the ripening time (*p* > 0.05, data not shown). Similarly, these parameters were not age-associated in Cheddar cheese, since fat distribution established in cheese at manufacturing remained unchanged across ripening [40]. Image analysis showed cheese porosity to increase significantly (*p* < 0.05, one-way ANOVA with respect to time) across ripening from 12 to 50 months. This finding is in agreement with data presented for Cheddar cheese where porosity was considered an effect of proteolysis progression after 30 weeks of ripening [41]. The CLSM images showed the presence of large calcium phosphate crystals in all samples (Figure 3, arrowheads), [11].

Similarly, numerous tyrosine crystals visible with the naked eye were present in 12mo cheeses (Table S2), in agreement with available literature [9,42]. In contrast, the white spots were absent in cheeses at this stage of ripening and appeared in increasing numbers subsequently. White spots are a typical feature of long-ripened hard cheeses, forming as a consequence of slow water migration within the cheese matrix that promotes compartmentalization of the most hydrophobic FAA in restricted areas [11]. This observation suggests that physico-chemical properties of cheese also evolved across an extremely long period.

Curves obtained from texture analysis showed the typical profile of extra-hard cheeses (data not shown). Changes in texture profile were monitored through measurement of both hardness and brittleness. The cheese hardness significantly (*p* < 0.05, one-way ANOVA with respect to time) increased with ripening time (Table 4), differences being significant after 30-month ripening with respect to 12 months. The brittleness instead, did not show a specific trend. Normally, cheese hardness decreases during early ripening as a consequence of proteolysis and hydration of the casein strands, both of which reduce the strength of the casein network. However, loss of moisture in turn causes an increase in protein concentration having an opposite effect [43]. Similar to our results, Noël et al. [9] observed firmness but not brittleness to increase with age in PR cheese during ripening from 12 to 28 months.

### 3.5. Cheese Color

Color analysis evidenced that the color of PR changed during the ripening period considered (Table 5). The magnitude of color difference between the color indexes recorded at each ripening time and that recorded at 12 months was estimated with ΔE* values which were all higher than 3, i.e., the threshold for a clearly perceivable color difference [44]. After 30 months of ripening, the difference increased due to the reduction of luminosity (L*) values that passed from an average value of 76 to 70 after 40 and 50 months. This trend could be due to the complex modifications taking place in PR cheese during ripening, especially the moisture reduction as shown in Table 1.

Yellowness index was not related to cheese age. This index is usually associated with seasonal variation of β-carotene and carotenoids content in the diet of dairy cattle [45], and we cannot exclude an initial difference in those components of our samples. Hue angle (h*) is another color index that can give an indication about the yellow character of the sample; in particular, an angle of 90° represents the yellow hue. While moving towards lower values, the color turns towards pale yellow up to green hue. Hue angle values at 12 and 50 months ripening were very close to 90° and statistically different from the other samples which correspond to lighter yellow hue, in accordance with YI values.

### 3.6. Principal Component Analysis

**Figure 4:**
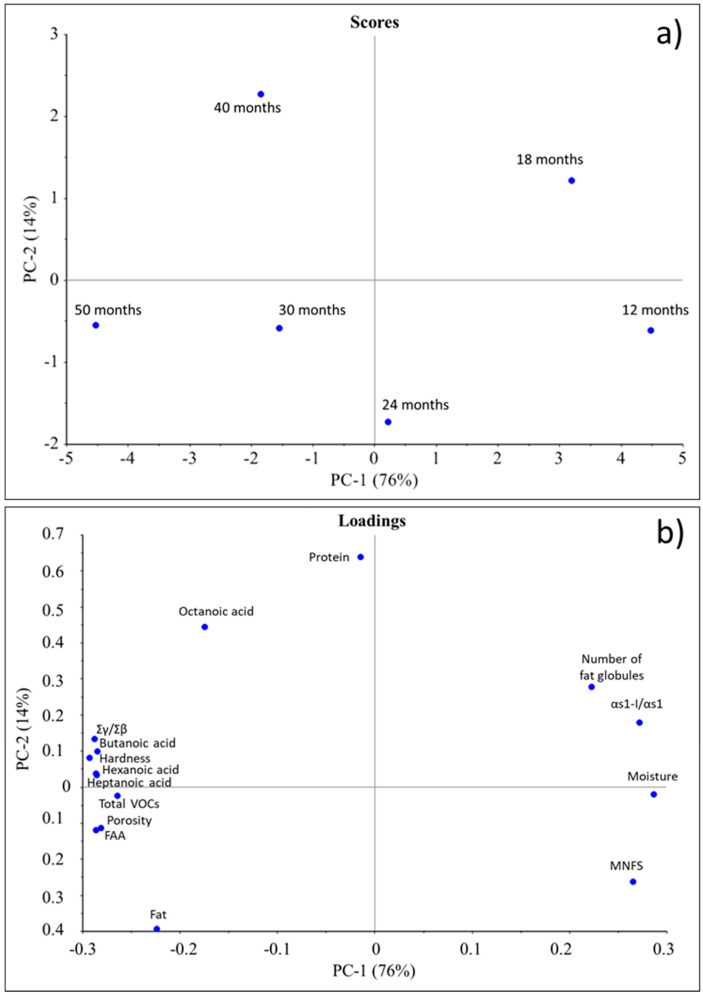
Principal component analysis (**a**), and loadings (**b**) of selected parameters evaluated in Parmigiano Reggiano cheeses ripened up to 50 months.

Principal component analysis (PCA) was performed to evaluate whether cheese samples were characterized by their ripening periods. A graphic display of loadings and scores is shown in Figure 4. Principal components 1 (PC1) and 2 (PC2) explained 76% and 14% of the total variance, respectively. Cheeses distributed along the PC1, with longer ripened samples (30–50 months) well distinguished from younger ones (12–24 months). Based on the loadings plot, two classes of variables grouped with a strong positive (moisture, αs1-I/ αs1, MNFS and number of fat globules) or a strong negative (Σγ/Σβ, butanoic acid, hexanoic acid, heptanoic acid, total VOCs, porosity, hardness and FAA) correlation with the ripening time. These two groups of variables were negatively correlated to each other. Detailed correlation coefficients between the variables are presented in Table S3. Butanoic, hexanoic and heptanoic acids showed a strong positive correlation (r = 0.988, *p* < 0.001; r = 1.000, *p* < 0.001; r = 0.882, *p* < 0.05, respectively) with the total sum of VOCs areas, while the contents of fat, protein and octanoic acid did not show a significant correlation with any other variables (*p* > 0.05).

## 4. Conclusions

Nowadays, the ripening time is being increased, especially for some hard and extra-hard cheese varieties, to produce premium quality products destined for target markets. Considering that increasing the ripening duration implies parallel increasing costs and, therefore, that these cheeses have a higher price, it was important to elucidate whether changes in cheese characteristics were actually taking place during the extra ripening period. Moisture was the component that changed the most during ripening, even between 40 and 50 months, and a strong negative correlation with cheese hardness was evidenced. In spite of the low water content, both primary and secondary proteolysis proceeded up to 50 months of ripening suggesting a pool of different enzymes still to be active. In parallel, the total volatile fraction increased with a strong contribution of short-chain FFA. Overall, this study showed for the first time that numerous biochemical and structural variations are still ongoing in a hard cheese up to 50 months, proving that the length of ripening deserves to be highlighted to consumers so that they can consciously buy a product with peculiar characteristics that support its premium price.

## Figures and Tables

**Table 1:** Gross composition (g/100 g) of Parmigiano Reggiano cheeses ripened from 12 to 50 months.

Ripening Time (Months)	Moisture	Protein	Fat	FDM^1^	MNFS^2^
12	31.72 ^c^ ± 0.07	31.17 ^a^ ± 0.07	30.78 ^ab^ ± 0.02	45.08 ^b^ ± 0.03	45.83 ^c^ ± 0.09
18	30.61 ^bc^ ± 2.42	33.72 ^d^ ± 0.15	29.23 ^a^ ± 2.33	42.08 ^a^ ± 1.87	43.20 ^b^ ± 2.02
24	29.39 ^bc^ ± 1.83	31.37 ^ab^ ± 0.32	32.61 ^bc^ ± 2.14	46.15 ^b^ ± 1.84	43.59 ^bc^ ± 1.32
30	28.86 ^abc^ ± 0.48	31.75 ^bc^ ± 0.16	32.48 ^bc^ ± 0.38	45.65 ^b^ ± 0.25	42.73 ^b^ ± 0.48
40	28.03 ^ab^ ± 0.31	33.79 ^d^ ± 0.11	30.43 ^ab^ ± 0.30	42.29 ^a^ ± 0.26	40.30 ^a^ ± 0.29
50	25.34 ^a^ ± 0.52	31.93 ^c^ ± 0.66	35.74 ^c^ ± 0.10	47.87 ^b^ ± 0.43	39.43 ^a^ ± 0.85

The results are expressed as the mean ± the standard deviation. ^a,b,c,d^ Mean values within a column with different superscripts are significantly different (*p* < 0.05; Tukey’s test). ^1^ FDM = fat in dry matter. ^2^ MNFS = moisture in nonfat substance.

**Table 2:** Casein fraction ratios (αs_1_-I/αs1, αs1 f(1-23)/ αs1 and Σγ/Σβ), calculated cheese age (months) and total free amino acids (FAA) (g/100 g protein) in Parmigiano Reggiano cheeses ripened from 12 to 50 months.

Ripening Time (Months)	αs_1_-I/αs_1_ (1)	αs_1_ f(1-23)/αs_1_ (2)	Σγ/Σβ (3)	Calculated Cheese Age (4)	FAA
12	0.7	0.4	2.7	13	24.2 ± 0.08 ^a^
18	0.6	0.3	5.0	22	24.6 ± 0.17 ^a^
24	0.3	0.2	5.4	25	28.4 ± 0.25 ^b^
30	0.3	0.2	8.7	34	28.6 ± 1.30 ^b^
40	0.4	0.3	10.0	39	28.4 ± 0.26 ^b^
50	0.2	0.2	12.1	49	30.0 ± 0.86 ^c^

The results are expressed as the mean ± standard deviation. ^(1)(2)(3)(4)^ Equations presented in Section 2.5. ^a,b,c^ Mean values within FAA column with different superscripts are significantly different (*p* < 0.05; Tukey’s test).

**Table 3:** Volatile compounds (area units/10,000) identified in Parmigiano Reggiano cheeses ripened from 12 to 50 months.

Compound	LRI ^a^	Identification ^b^	Ripening Time (Months)					
			12	18	24	30	40	50
*Acids*								
Acetic acid	1455	MS, PI	1000 ± 200	850 ± 270	950 ± 60	1300 ± 120	1100 ± 110	1200 ± 90
Propanoic acid	1546	MS, PI	3 ± 0	24 ± 2	4 ± 2	7 ± 1	8 ± 1	6 ± 1
Butanoic acid	1640	MS, PI	4300 ± 300	4500 ± 700	5500 ± 300	8100 ± 500	9300 ± 400	11000 ± 890
Pentanoic acid	1755	MS, PI	18 ± 1	21 ± 4	41 ± 14	73 ± 8	97 ± 9	91 ± 7
Hexanoic acid	1870	MS, PI	4800 ± 900	5600 ± 760	7000 ± 700	12000 ± 1200	12000 ± 890	17000 ± 1300
Heptanoic acid	1957	MS, PI	ND	6 ± 3	28 ± 21	74 ± 50	23 ± 15	58 ± 23
Octanoic acid	2066	MS, PI	ND	71 ± 10	170 ± 60	1300 ± 1200	500 ± 67	1700 ± 500
2-Methylbutanoic acid		T	4 ± 0.3	4 ± 0.3	3 ± 0.5	2 ± 0.4	3 ± 0.2	3 ± 0.2
3-Methylbutanoic acid		T	40 ± 0.3	36 ± 5	16 ± 5	20 ± 3	6 ± 1	20 ± 3
*Sum of areas*			*10165*	*11112*	*13712*	*22876*	*23037*	*31078*
*Ketones*								
2-Pentanone	990	MS, PI, ST	150 ± 12	400 ± 90	400 ± 110	300 ± 70	300 ± 50	340 ± 50
2-Heptanone	1190	MS, PI, ST	200 ± 40	570 ± 180	700 ± 40	600 ± 130	600 ± 70	630 ± 120
2-Nonanone	1400	MS, PI, ST	80 ± 2	170 ± 40	400 ± 100	290 ± 100	270 ± 60	700 ± 200
8-Nonen-2-one	1495	MS, PI	ND	15 ± 2	30 ± 15	40 ± 4	20 ± 4	70 ± 21
*Sum of areas*			*430*	*1155*	*1530*	*1230*	*1190*	*1740*
*Alcohols*								
2-Pentanol	1172	MS, PI, ST	95 ± 8	97 ± 76	86 ± 83	45 ± 8	18 ± 4	48 ± 18
2-Heptanol	1320	MS, PI	45 ± 4	33 ± 23	38 ± 23	17 ± 2	17 ± 4	37 ± 17
*Sum of areas*			*140*	*130*	*124*	*62*	*35*	*85*
*Esters*								
Ethyl butanoate	1055	MS, PI	250 ± 1	440 ± 49	150 ± 14	580 ± 96	330 ± 96	500 ± 87
Ethyl hexanoate	1240	MS, PI	430 ± 54	1000 ± 170	230 ± 70	2000 ± 75	720 ± 132	1300 ± 170
Ethyl octanoate	1440	MS, PI	35 ± 2	98 ± 8	11 ± 4	287 ± 35	81 ± 17	249 ± 30
*Sum of areas*			*715*	*1538*	*391*	*2867*	*1131*	*2049*
*Total sum of areas*			*11450*	*13935*	*15757*	*27035*	*25393*	*34952*

Data are expressed as the mean ± standard deviation. ^a^ Linear Retention Index in cheese samples using a StabilWax Column. ^b^ Identification method: MS = identification by spectra comparison in NIST Library; PI = comparison with published LRI; T = tentatively identified; ST = standard injection.

**Table 4:** Image analysis parameters (number of fat globules and porosity), hardness (N) and brittleness (mm) of Parmigiano Reggiano cheeses ripened from 12 to 50 months.

Ripening Time (Months)	Number of Fat Globules	Porosity	Hardness	Brittleness
12	676 ± 28 ^c^	0.11 ± 0.01 ^a^	11.96 ± 1.57 ^a^	5.02 ± 0.34 ^a^
18	574 ± 149 ^bc^	0.11 ± 0.01 ^a^	15.98 ± 0.29 ^abc^	6.31 ± 0.38 ^ab^
24	292 ± 96 ^a^	0.17 ± 0.01 ^b^	18.93 ± 0.49 ^abcd^	6.52 ± 0.82 ^b^
30	410 ± 114 ^abc^	0.16 ± 0.01 ^b^	21.48 ± 0.49 ^bcd^	6.30 ± 0.94 ^ab^
40	476 ± 65 ^abc^	0.17 ± 0.01 ^b^	22.65 ± 4.31 ^cbcd^	6.52 ± 0.69 ^ab^
50	363 ± 150 ^ab^	0.19 ± 0.01 ^c^	25.20 ± 3.53 ^d^	5.53 ± 0.69 ^a^

The results are expressed as the mean ± standard deviation. ^a,b,c,d^ Mean values within a column with different superscripts are significantly different (*p* < 0.05; Tukey’s test). Number of fat globules and porosity were calculated in a volume sample of 212 µm × 212 µm × 7 µm.

**Table 5:** Color parameters of Parmigiano Reggiano cheeses ripened from 12 to 50 months.

Ripening Time (Months)	ΔE* (6)	YI (5)	h* (7)
12		35.07 ^a^ ± 0.62	88.65 ^a^ ± 0.19
18	3.66 ^a^ ± 0.74	31.42 ^a^ ± 1.32	82.75 ^bc^ ± 0.31
24	3.26 ^a^ ± 1.22	31.04 ^a^ ± 2.97	82.69 ^bc^ ± 1.62
30	6.72 ^b^ ± 1.31	26.28 ^b^ ± 2.94	80.57 ^c^ ± 1.07
40	7.73 ^b^ ± 0.91	26.24 ^b^ ± 3.02	84.80 ^b^ ± 0.55
50	6.38 ^b^ ± 1.50	31.40 ^a^ ± 2.14	87.64 ^a^ ± 2.85

The results are expressed as the mean ± standard deviation. ^(5)(6)(7)^ Equations presented in Section 2.5. ^a,b,c^ Mean values within a column with different superscripts are significantly different (*p* < 0.05; Tukey’s test). ΔE* = total color difference; YI = yellowness index; h* = hue angle.

## Supplementary Materials

The following are available online at https://www.mdpi.com/2304-8158/9/3/268/s1, Figure S1: Parmigiano Reggiano manufacture process; Table S1: Free amino acid composition of Parmigiano Reggiano cheeses ripened up to 50 months; Table S2: Number of tyrosine crystals and spots counted in Parmigiano Reggiano cheeses ripened up to 50 months; Table S3: Correlation coefficient matrix.
