# Supplementary material for: Impact of Extending Hard-Cheese Ripening: A Multiparameter Characterization of Parmigiano Reggiano Cheese Ripened up to 50 Months
Source: Foods. 2020 Mar 2;9(3):268. doi: 10.3390/foods9030268 (PMC7143483; doi:10.3390/foods9030268)
Supplement: Supplementary file 1 [file foods-09-00268-s001.pdf]

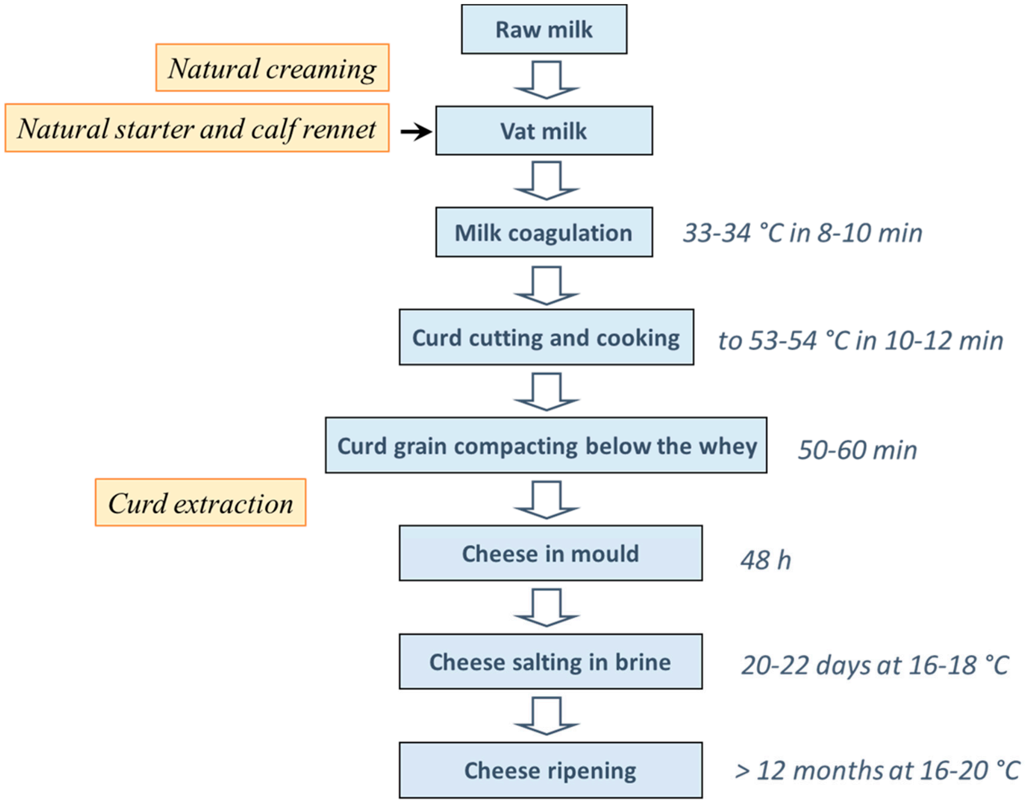

Figure S1 Parmigiano Reggiano manufacture process and ripening conditions adopted at the cheese factories involved in this study.

Table S1. Free amino acid (FAA) composition (g/100 g FAA) of Parmigiano-Reggiano cheeses ripened up to 50 months. Results are expressed as mean ± standard deviation.

Ripening time (months)	Asp	Thr	Ser	Asn	Glu	Gln	Gly	Ala	Cit	Val	Met	Ile	Leu	Tyr	Phe	Gaba	Orn	Lys	His	Arg	Pro
12	3.86 ± 0.03	3.52 ± 0.05	6.01 ± 0.05	4.05 ± 0.03	17.04 ± 0.26	0.81 ± 0.04	2.25 ± 0.03	2.62 ± 0.03	3.31 ± 0.05	7.33 ± 0.05	2.32 ± 0.06	6.26 ± 0.26	8.52 ± 0.12	3.6 ± 0.19	4.96 ± 0.08	0 ± 0	0.9 ± 0.02	10.3 ± 0.05	2.66 ± 0.04	0.67 ± 0.01	9 ± 0.16
18	4.44 ± 0.56	2.99 ± 0.06	6.36 ± 0.12	3.33 ± 0.44	16.76 ± 0.33	0.4 ± 0.04	2.64 ± 0.11	2.84 ± 0.05	3.37 ± 0.17	7.56 ± 0.22	2.33 ± 0.06	6.46 ± 0.12	8.18 ± 0.18	3.37 ± 0.14	4.67 ± 0.23	0.2 ± 0.22	0.96 ± 0.08	10.68 ± 0.17	2.77 ± 0.14	0.24 ± 0.27	9.44 ± 0.20
24	4.2 ± 0.42	3.8 ± 0.07	6.92 ± 0.06	3.83 ± 0.36	16.16 ± 0.42	0.15 ± 0.16	2.16 ± 0.03	2.69 ± 0.04	0.85 ± 0.08	7.67 ± 0.18	2.37 ± 0.06	6.38 ± 0.09	7.99 ± 0.27	3.32 ± 0.25	4.47 ± 0.13	0.53 ± 0.24	0.16 ± 0.06	10.36 ± 0.16	2.86 ± 0.02	3.65 ± 0.39	9.46 ± 0.27
30	4.18 ± 0.1	2.2 ± 0.11	7.12 ± 0.28	3.83 ± 0.06	16.6 ± 0.49	0.07 ± 0.07	3.28 ± 0.15	2.89 ± 0.09	2.47 ± 0.15	7.73 ± 0.24	2.65 ± 0.26	6.69 ± 0.39	8.43 ± 0.44	2.78 ± 1.09	4.68 ± 0.07	0 ± 0	1.52 ± 0.03	10.63 ± 0.62	2.31 ± 0.04	0 ± 0	9.96 ± 0.30
40	4.8 ± 0.24	3.74 ± 0.06	7.53 ± 0.05	3.52 ± 0.03	16.7 ± 0.1	0 ± 0	2.26 ± 0.07	2.71 ± 0.02	2.3 ± 0.75	7.63 ± 0.22	2.28 ± 0.14	6.3 ± 0.13	7.72 ± 0.65	2.88 ± 0.36	4.63 ± 0.06	0.1 ± 0.11	0.34 ± 0.07	10.48 ± 0.13	2.61 ± 0.05	2.06 ± 1.12	9.42 ± 0.21
50	7.11 ± 0.11	2.77 ± 0.07	7.48 ± 0.20	0.73 ± 0.02	16.16 ± 0.5	0 ± 0	2.71 ± 0.09	2.75 ± 0.06	2.11 ± 0.02	7.55 ± 0.11	2.83 ± 0.37	7.21 ± 0.72	9.01 ± 0.39	2.96 ± 0.16	4.46 ± 0.16	0.64 ± 0.15	1.81 ± 0.09	10.13 ± 0.34	2.24 ± 0.05	0 ± 0	9.36 ± 0.32

Table S2. Number of tyrosine crystals and spots counted on a surface of 100 cm^2 in Parmigiano-Reggiano cheeses ripened up to 50 months.

Ripening time (months)	Tyrosine crystals	Spots
12	92	0
18	42	182
24	53	415
30	98	332
40	55	277
50	59	590

Table S3. Correlation coefficients between compositional, rheological and chemical parameters. The asterisks indicate the significance levels (*$p < 0.05$; ** $p < 0.01$; ***$p < 0.001$).

	Moisture	Fat	Protein	MNFS	Hardness	FAA	αs1-I/αs1	Σγ/Σβ	Number of fat globules	Porosity	Butanoic acid	Hexanoic acid	Heptanoic acid	Octanoic acid	Total VOCs
Moisture	1.000														
Fat	−0.782	1.000													
Protein	−0.089	−0.534	1.000												
MNFS	0.929*	−0.498	−0.445	1.000											
Hardness	−0.950**	0.651	0.197	−0.931*	1.000										
FAA	−0.890*	0.769	−0.102	−0.773	0.939*	1.000									
αs1-I/αs1	0.837	−0.755	0.126	0.708	−0.904*	−0.980**	1.000								
Σγ/Σβ	−0.963**	0.655	0.219	−0.950**	0.972**	0.866*	−0.803	1.000							
Number of fat globules	0.672	−0.674	0.197	0.526	−0.744	−0.877*	0.945*	−0.588	1.000						
Porosity	−0.891*	0.768	−0.105	−0.775	0.916*	0.988***	−0.956**	0.840	−0.867*	1.000					
Butanoic acid	−0.952**	0.701	0.108	−0.908*	0.934*	0.858*	−0.767	0.981**	−0.530	0.844*	1.000				
Hexanoic acid	−0.952**	0.756	0.040	−0.875*	0.932*	0.863*	−0.794	0.979**	−0.565	0.832*	0.987**	1.000			
Heptanoic acid	−0.762	0.611	−0.036	−0.687	0.875*	0.873*	−0.845	0.858*	−0.652	0.801	0.848	0.880*	1.000		
Octanoic acid	−0.518	0.012	0.526	−0.700	0.634	0.518	−0.390	0.636	−0.194	0.544	0.658	0.546	0.535	1.000	
Total VOCs	−0.954**	0.753	0.046	−0.881*	0.938*	0.870*	−0.801	0.981**	−0.574	0.840*	0.988***	1.000***	0.882*	0.556	1.000
